# Frailty as a Key Determinant of In-Hospital Mortality in 58,040 Patients with Community-Acquired Pneumonia: Evidence from a Chilean Cohort

**DOI:** 10.3390/jcm15041442

**Published:** 2026-02-12

**Authors:** Yeny Concha-Cisternas, Manuel Vásquez-Muñoz, Rodrigo Yañez-Sepúlveda, Sergio Sazo-Rodríguez, Felipe Diaz Canales, Christopher Fuentes Orellana, Patricia Schonffeldt, Eduardo Guzmán-Muñoz

**Affiliations:** 1 Escuela de Kinesiología, Facultad de Salud, Universidad Santo Tomás, Talca 3460000, Chile; sergiosazoro@santotomas.cl (S.S.-R.); eduardo.guzman@cloud.uautonoma.cl (E.G.-M.); 2 Vicerrectoría de Investigación e Innovación, Universidad Arturo Prat, Iquique 1100000, Chile; 3 Center for Health Data Observation and Analysis (CADS), School of Medicine and Health Sciences, Universidad Mayor, Santiago 8580745, Chile; 4 Escuela de Medicina, Facultad de Medicina y Ciencias de la Salud, Universidad Mayor, Santiago 8580745, Chile; 5 Facultad de Educación y Ciencias Sociales, Universidad Andrés Bello, Viña del Mar 2200055, Chile; rodrigo.yanez.s@unab.cl; 6 School of Medicine, Universidad Espíritu Santo, Samborondón 092301, Ecuador; 7 Servicio Medicina Física y Rehabilitación, Hospital Regional de Talca, Talca 3460000, Chile; fdiazc@hospitaldetalca.cl; 8 Departamento Comunal de Salud Maule, Maule 3530000, Chile; cfuentes@csmaule.com; 9 Instituto Nacional del Tórax, Santiago 8580745, Chile; schonffeldtpatricia@gmail.com; 10 Atrys Broncopulmonar, Santiago 8580745, Chile; 11 Escuela de Pedagogía en Educación Física, Facultad de Educación, Universidad Autónoma de Chile, Talca 3460000, Chile

**Keywords:** pneumonia, frailty, hospital mortality, hospital frailty risk score

## Abstract

**Background:** Pneumonia is a leading cause of hospitalization and death among older adults, reflecting both patient vulnerability and the quality of acute care. However, evidence from Latin America remains limited. **Objective:** to examine the association between frailty level assessed using the HFRS and in-hospital mortality among older adults hospitalized with community-acquired pneumonia (CAP). **Methods:** We conducted a retrospective cohort study using the Chilean National Health Fund (Fondo Nacional de Salud, FONASA) database, including patients aged ≥ 60 years hospitalized with CAP (ICD-10 codes J12–J18) between 2019 and 2024. Variables analyzed included age, sex, frailty level assessed by the Hospital Frailty Risk Score (HFRS), comorbidity burden (Charlson Comorbidity Index), Diagnosis-Related Group (DRG) severity level, and relative weight. Survival was analyzed using Kaplan–Meier curves and log-rank tests. Multivariable Cox proportional hazards models estimated adjusted hazard ratios (HR) with 95% confidence intervals (CI). **Results:** The cohort comprised 58,040 patients (51.2% women). Overall, in-hospital mortality was 19.3%. Independent predictors of mortality included advanced age (≥90 years: HR = 2.41; 95% CI: 2.27–2.56), male sex (HR = 1.10; 95% CI: 1.06–1.14), high frailty risk (HR = 1.57; 95% CI: 1.47–1.68), and greater DRG severity (per level: HR = 1.66; 95% CI: 1.58–1.73). The Charlson Comorbidity Index lost significance after adjustment. **Conclusions:** Frailty emerged as a strong and independent determinant of in-hospital mortality in older adults with CAP. Systematic frailty assessment combined with comorbidity indices could improve risk stratification and guide more personalized, evidence-based clinical management in acute care settings.

## 1. Introduction

Pneumonia in older adults represents a major global burden of morbidity and mortality, ranking among the leading causes of death from respiratory diseases [1,2]. Global Burden of Disease (GBD) 2019 estimates indicate that lower respiratory tract infections, including pneumonia, remained a major contributor to global morbidity and mortality before the COVID-19 pandemic [3]. Globally, lower respiratory tract infections accounted for approximately two million deaths annually [3], nearly 50% of these occurring in individuals aged 70 years or older [4]. In this context, community-acquired pneumonia (CAP) is one of the leading causes of hospitalization and mortality among older adults, and as population aging continues to progress, it is expected to become an increasingly critical public health issue [5]. Worldwide, pneumonia remains a major cause of morbidity and mortality among older adults. Recent post-pandemic evidence has shown wide variability in mortality outcomes, ranging from 3.5% to 17.7% in the United States and 13% [6] to 21.5% in Europe [7,8,9]. In contrast, in Latin America, pre-pandemic studies reported in-hospital mortality rates between 10% and 35% [10] and updated post-COVID data are still lacking. Similarly, other investigations have documented mortality rates reaching up to 39% in older patients, further underscoring the marked vulnerability of this population [11]. In Chile, pneumonia-related mortality among older adults reached 22.2%, increasing substantially with advancing age [12]. This rise may be attributed to the presence of multiple chronic comorbidities and the progressive decline in immune function associated with aging, both of which have a direct impact on clinical prognosis [7,9]. Although international evidence provides a robust understanding of the burden and prognosis of community-acquired pneumonia in older adults, data from Latin America—particularly Chile—remain limited despite the region’s rapid demographic aging and high prevalence of chronic conditions. This gap highlights the need for locally derived evidence to better contextualize pneumonia outcomes and support clinical and policy decision-making in the Chilean healthcare setting.

Frailty has emerged as a key determinant of hospital outcomes in older adults with pneumonia. It is defined as a state of increased vulnerability resulting from diminished physiological reserves and a reduced ability to respond to acute stressors, which heightens the risk of adverse events [13,14]. Although frailty prevalence varies across hospitalized populations depending on the assessment tool and clinical context [15,16,17], it may reach up to 66.8% among older adults admitted with CAP [18]. A recent systematic review and meta-analysis similarly reported that approximately half of older adults hospitalized with pneumonia are frail and that frailty is independently associated with higher in-hospital mortality, longer hospital stays, increased readmission risk, and poorer functional recovery [19]. Evidence from Asian cohorts also demonstrates the prognostic importance of frailty. In Korea, frail older adults hospitalized with pneumonia had substantially higher rates of the composite outcome of 30-day death or functional decline compared with non-frail patients (75% vs. 52%) [20], while a prospective study from China showed that frailty conferred nearly a threefold higher one-year mortality risk even after adjusting for comorbidities and pneumonia severity [18]. Despite this robust international evidence, the systematic integration of frailty assessment into clinical practice and risk stratification models remains limited in Latin America and is virtually absent in Chile.

This gap is further compounded by the fact that, although frailty is widely acknowledged as a critical prognostic factor, most hospitals continue to use conventional risk-adjustment systems such as the Charlson Comorbidity Index or Elixhauser Comorbidity Index to estimate comorbidity burden, administrative severity, and in-hospital mortality [21,22]. However, these conventional tools are primarily based on diagnostic and administrative data and may fail to capture key clinical and functional aspects of patients particularly those related to biological vulnerability and frailty. In this context, the Hospital Frailty Risk Score (HFRS), developed from administrative health records and validated across various healthcare systems, provides an objective, standardized, and low-cost method to identify frail patients who are at increased risk of adverse outcomes [23,24]. Incorporating systematic frailty screening through appropriate tools in patients hospitalized with CAP could improve the predictive accuracy of risk models, help identify subgroups with greater biological vulnerability, and guide more precise and cost-effective clinical strategies [25,26]. Given these considerations, the present study aimed to examine the association between frailty level assessed using the HFRS and in-hospital mortality among older adults hospitalized with CAP. We hypothesized that higher frailty levels, as quantified by the HFRS, would be independently associated with an increased risk of in-hospital mortality among Chilean older adults hospitalized with community-acquired pneumonia.

## 2. Materials and Methods

This retrospective cohort study analyzed data from the public database of the Chilean National Health Fund (Fondo Nacional de Salud, FONASA), which compiles anonymized hospital discharge records from all public hospitals in Chile. The analysis included records from 72 public hospitals that are part of the national healthcare network administered by FONASA. All of these institutions are medium- and high-complexity centers with the capacity to manage acute respiratory conditions and provide multidisciplinary care for older adult patients.

### 2.1. Data Collection

**Figure 1:**
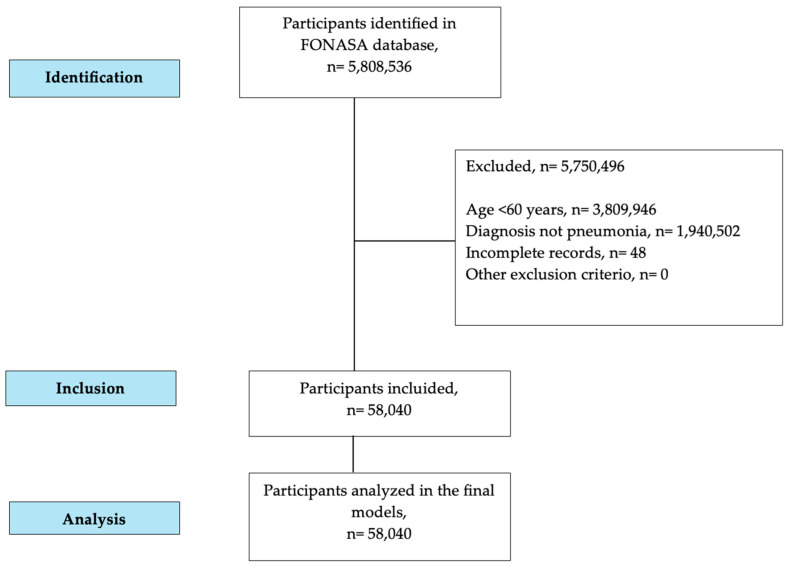
STROBE flowchart of participants.

Hospital discharge records were obtained from the publicly available database of the FONASA https://datosabiertos.fonasa.cl (accessed on 11 September 2025) which compiles anonymized information from all public hospitals in the country. All hospitalizations of adults aged 60 years and older with a principal diagnosis of CAP recorded in the FONASA database between 1 January 2019, and 31 December 2024, were included (Figure 1). CAP was identified using the International Classification of Diseases, 10th Revision (ICD-10) codes J12–J18, encompassing viral, bacterial, and unspecified pneumonias. COVID-19 pneumonias were excluded using SARS-CoV-2–specific ICD-10 codes; however, the FONASA administrative dataset does not include standardized information on SARS-CoV-2 testing practices or routine COVID-19 rule-out protocols across hospitals, making it impossible to confirm whether COVID-19 etiology was systematically excluded at the clinical level during the study period. In Chile, ICD-10 coding is part of a standardized national diagnostic framework used across all public hospitals and routinely applied in epidemiological studies of respiratory infections. Although formal validation studies of ICD-10 coding for CAP are limited, the coding system is mandatory and subject to periodic auditing, supporting its reliability for large-scale administrative analyses.

The FONASA database includes standardized fields containing demographic, clinical, and administrative information, as well as hospital outcomes (discharged alive or deceased). All variables analyzed in this study were extracted directly from these standardized records, thus eliminating the need for individual chart review or additional validation through imaging or clinical reports. Mortality data referred exclusively to in-hospital deaths recorded at the time of discharge, as no post-discharge follow-up was conducted. Records were excluded when information on age, sex, or discharge status was missing, when diagnoses fell outside the specified ICD codes, or when cases involved patient transfers or duplicate admissions. The proportion of missing data was minimal; therefore, incomplete records were removed to maintain dataset integrity and ensure analytic consistency. Data validation procedures included verifying the consistency of admission and discharge identifiers to ensure completeness, avoid duplication, and confirm the reliability of hospitalization records.

### 2.2. Variables

#### 2.2.1. Dependent Variable

The dependent variable was in-hospital mortality, recorded in the FONASA hospital discharge database as the discharge condition (0 = survived, 1 = deceased). Time-to-event was defined as the length of hospital stay in days, calculated from the date of admission to the date of discharge or death. Mortality data correspond exclusively to deaths occurring during hospitalization; no post-discharge follow-up was performed.

#### 2.2.2. Independent Variables

The independent variables extracted from the FONASA database were as follows: (i) Sociodemographic variables: Sex (male or female) and age (analyzed as a continuous variable and categorized into age groups 60–69, 70–79, 80–89, and ≥90 years); (ii) Clinical and administrative variables: (a) Frailty risk; (b) Severity level: obtained from the Chilean Diagnosis-Related Groups (DRG) classification, ranging from mild to severe, based on expected resource use and clinical complexity, (c) Relative weight: a unitless index from the DGR system representing the expected resource consumption for each hospitalization relative to the national average and (d) Charlson comorbidity weight.

Frailty was classified using the HFRS, a low-cost risk stratification tool that does not require direct clinical assessment but instead relies on routinely collected electronic medical records [23,24,27]. This index was calculated based on 109 diagnostic categories from the ICD-10, identified in each patient and selected for their association with conditions frequently observed in frail individuals and adverse clinical outcomes. Each diagnosis carries a specific weight, and the total weighted sum represents the individual frailty score. Patients were classified into three risk categories: low frailty risk (<5 points), intermediate frailty risk (5–15 points), and high frailty risk (>15 points). The HFRS was computed using the ICD-10 codes recorded during hospitalization, following the methodological criteria described by Gilbert et al. (2018) [23] and subsequently replicated by Eckart et al. (2019) [24].

In Chile, DRG severity level is an administrative classification that reflects the expected clinical complexity and resource utilization of each hospitalization. It is derived from diagnostic and procedural information recorded in the discharge summary and is widely used for hospital management, cost estimation, and case-mix adjustment within the national health system. However, clinical pneumonia severity scores such as the Pneumonia Severity Index (PSI) and CURB-65 are not routinely recorded in administrative datasets and were therefore unavailable in the FONASA database. Unlike these clinical indices, which incorporate physiological, laboratory, and functional parameters, the DRG severity level serves as an administrative proxy of hospitalization complexity and does not directly measure biological or physiological severity. This distinction should be considered when comparing our findings with international CAP cohorts that rely on PSI or CURB-65 for risk stratification.

Comorbidity burden was assessed using the Charlson Comorbidity Index, a validated instrument designed to quantify the overall chronic disease load and estimate mortality risk based on coded clinical diagnoses. The Charlson Comorbidity Index comprises 17 chronic conditions, each assigned a specific weight ranging from 1 to 6 points according to its strength of association with one-year mortality [21,28]. In this study, comorbidities were identified using ICD-10 codes, which were mapped to the corresponding Charlson Comorbidity Index categories according to the algorithms proposed by Quan et al. (2005) for administrative health data [29]. Each ICD-10 code was assigned to its corresponding weighted disease category, and the total Charlson Comorbidity Index score was obtained by summing all applicable weights for each patient. Higher Charlson Comorbidity Index values indicate a greater cumulative comorbidity burden and, consequently, a higher baseline risk of in-hospital mortality.

### 2.3. Statistical Analysis

Statistical analyses were performed using GraphPad Prism, version 9 (GraphPad Software, San Diego, CA, USA). The analysis of in-hospital mortality was conducted in two stages: descriptive and analytical. In the descriptive stage, continuous variables were expressed as mean and standard deviation (SD), while categorical variables were summarized as absolute and relative frequencies.

For the analytical stage, time-to-event analysis was performed using the Kaplan–Meier method to estimate survival functions according to age group, sex, frailty risk, and DRG severity level. Differences between survival curves were assessed using the log-rank (Mantel–Cox) test, and the Gehan–Breslow–Wilcoxon test was additionally reported to provide greater sensitivity to early in-hospital events. Median survival times were calculated and reported when applicable.

Cox proportional hazards regression models were used to estimate unadjusted and adjusted hazard ratios (HR) with their corresponding 95% confidence intervals (CI). In the univariate stage, each variable was analyzed separately. The multivariable model included age group, sex, frailty risk, DRG severity, Charlson comorbidity weight, and relative weight. The proportional hazards assumption was verified for all covariates included in the final model. Kaplan–Meier survival curves were generated for the main variables that remained statistically significant in the adjusted Cox model. The proportional hazards assumption was verified for all covariates using Schoenfeld residuals and global tests. Linearity of continuous predictors was assessed by examining Martingale residuals. For all analyses, a *p*-value < 0.05 was considered statistically significant.

## 3. Results

A total of 58,040 patients hospitalized with CAP were included in the study. Table 1 describes baseline characteristics of the study population. The mean age was 77.8 years (SD 9.7), and the sex distribution was nearly balanced, with 29,700 females (51.2%) and 28,340 males (48.8%). In terms of hospital outcomes, 11,199 patients (19.3%) died during hospitalization, whereas 46,841 (80.7%) were discharged alive. Regarding frailty risk, as assessed by the HFRS, 50.5% of patients were classified as intermediate risk, while 24.6% were categorized as low risk and 24.9% as high risk. The prevalence of in-hospital mortality varied across study years, reaching 18.0% in 2019, 22.1% in 2020, 24.5% in 2021, 21.6% in 2022, 18.0% in 2023, and 17.6% in 2024.

### 3.1. Survival Analyses

**Figure 2:**
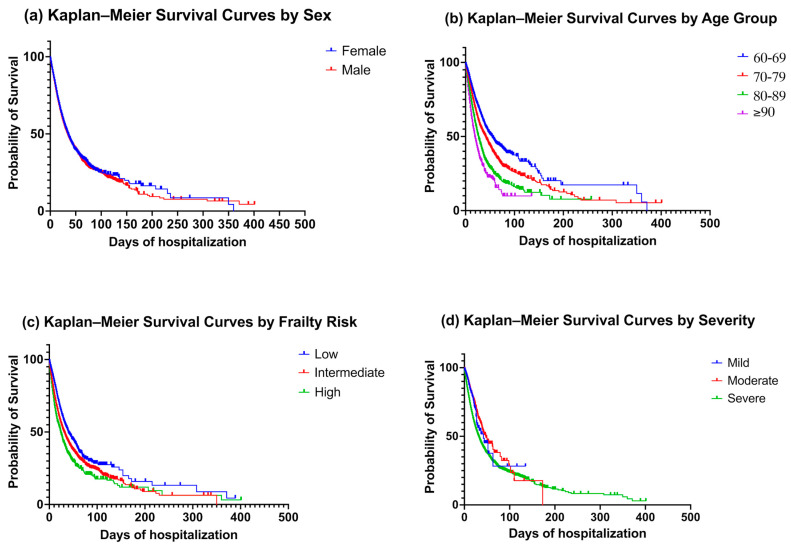
Kaplan–Meier survival curves for in-hospital mortality according to baseline characteristics. (**a**) Survival by sex (female vs. male); (**b**) survival by age group (60–69, 70–79, 80–89, and ≥90 years); (**c**) survival by frailty risk (low, intermediate, high, according to HFRS); and (**d**) survival by DRG severity level (mild, moderate, severe). Significant differences were observed across age groups, frailty risk categories, and severity levels (all log-rank *p* < 0.001), while sex showed only a marginal difference (*p* = 0.035). Median survival declined progressively with advancing age, higher frailty, and greater severity, highlighting their prognostic relevance in hospitalized patients with pneumonia.

Kaplan–Meier survival curves showed significant differences across sex, age group, frailty risk, and DRG severity level. When stratified by sex, men exhibited a slightly shorter median survival compared with women (34 vs. 35 days) (Figure 2a). Although the absolute difference was modest, both the log-rank test (χ^2^ = 4.43, df = 1, *p* = 0.035) and the Gehan–Breslow–Wilcoxon test (χ^2^ = 4.69, *p* = 0.030) indicated that male patients experienced a small but consistent survival disadvantage during hospitalization.

Marked differences in survival were also observed across age groups. Median survival declined progressively with advancing age: 55 days in patients aged 60–69 years, 41 days in those 70–79 years, 27 days in those 80–89 years, and only 19 days in patients aged ≥90 years (Figure 2b). The log-rank test confirmed these differences (χ^2^ = 1048, df = 3, *p* < 0.0001), and the log-rank test for trend supported a graded increase in mortality risk with older age (χ^2^ = 1011, *p* < 0.0001). Similarly, the Gehan–Breslow–Wilcoxon test (χ^2^ = 759.4, df = 3, *p* < 0.0001) emphasized that the detrimental effect of age was already evident in early hospital mortality.

Survival curves according to frailty risk demonstrated a clear and progressive decline from the low- to the high-risk categories. Median survival decreased from 40 days in the low-risk group to 31 days in the intermediate-risk group and 24 days in the high-risk group (Figure 2c). The log-rank test confirmed highly significant differences between categories (χ^2^ = 517.7, df = 2, *p* < 0.0001), while the log-rank test for trend supported a strong linear association between increasing frailty and decreasing survival (χ^2^ = 514.6, *p* < 0.0001). These findings were reinforced by the Gehan–Breslow–Wilcoxon test (χ^2^ = 559.3, df = 2, *p* < 0.0001), suggesting that frailty exerts its impact not only on overall survival but also on early in-hospital mortality.

Finally, survival curves stratified by DRG severity level also showed clear separation. Patients with mild severity had a median survival of 42 days, those with moderate severity had the longest survival at 48 days, and those with severe DRG presented the lowest median survival with 32 days (Figure 2d). Statistical testing corroborated these differences, as the log-rank test (χ^2^ = 590.6, df = 2, *p* < 0.0001), the log-rank test for trend (χ^2^ = 514.8, *p* < 0.0001), and the Gehan–Breslow–Wilcoxon test (χ^2^ = 595.1, df = 2, *p* < 0.0001) all demonstrated strong and consistent associations between higher severity and worse in-hospital survival.

### 3.2. Cox Regression Models

Table 2 presents the unadjusted and adjusted hazard ratios for in-hospital mortality. In the univariate analyses, older age was strongly associated with increased mortality, with HRs rising progressively across age groups: 70–79 years (HR 1.33, 95% CI 1.26–1.40, *p* < 0.001), 80–89 years (HR 1.86, 95% CI 1.77–1.97, *p* < 0.001), and ≥90 years (HR 2.59, 95% CI 2.43–2.76, *p* < 0.001), all compared with the 60–69 reference group. Male sex showed a small increase in risk, although it did not reach statistical significance (HR 1.04, 95% CI 1.00–1.08, *p* = 0.054). Frailty risk demonstrated a clear gradient, with intermediate (HR 1.42, 95% CI 1.37–1.48, *p* < 0.001) and high risk (HR 1.77, 95% CI 1.66–1.90, *p* < 0.001) both significantly increasing mortality compared with the low-risk group. Additional predictors included comorbidity burden (HR 1.01 per unit, 95% CI 1.00–1.02, *p* = 0.046) and DRG severity (HR 1.63 per level, 95% CI 1.56–1.70, *p* < 0.001). In contrast, relative weight was not significantly associated with mortality (HR 0.94 per unit, 95% CI 0.93–0.95, *p* = 0.101).

In the multivariable adjusted model, which included age, sex, frailty risk, DRG severity, Charlson comorbidity weight, and relative weight, most predictors remained significantly associated with in-hospital mortality. Age continued to be a strong determinant, with hazards increasing progressively across categories: 70–79 years (HR 1.28, 95% CI 1.22–1.35, *p* < 0.001), 80–89 years (HR 1.74, 95% CI 1.65–1.83, *p* < 0.001), and ≥90 years (HR 2.41, 95% CI 2.27–2.56, *p* < 0.001), all compared with the 60–69 reference group. Male sex, which was not significant in the univariate analysis, emerged as an independent predictor after adjustment (HR 1.10, 95% CI 1.06–1.14, *p* < 0.001). Frailty risk also retained a strong and independent association, with intermediate frailty increasing the hazard by 34% (HR 1.34, 95% CI 1.29–1.39, *p* < 0.001) and high frailty by 57% (HR 1.57, 95% CI 1.47–1.68, *p* < 0.001), both compared with the low-risk group. DRG severity was confirmed as an additional strong predictor (HR 1.66 per level, 95% CI 1.58–1.73, *p* < 0.001). In contrast, Charlson comorbidity weight was no longer statistically significant after adjustment (HR 0.99, 95% CI 0.98–1.00, *p* = 0.198). Finally, relative weight did not show a statistically significant association with mortality in the adjusted model (HR 0.94 per unit, 95% CI 0.93–0.95, *p* = 0.091).

## 4. Discussion

In this nationwide cohort of 58,040 patients admitted with a primary diagnosis of CAP, the in-hospital mortality rate was 19.3%. Multivariable Cox regression identified advanced age (HR 2.41; 95% CI 2.27–2.56), male sex (HR 1.10; 95% CI 1.06–1.14), high frailty risk (HR 1.57; 95% CI 1.47–1.68), and higher DRG severity (per-level HR 1.66; 95% CI 1.58–1.73) as independent predictors of mortality, while the Charlson Comorbidity Index lost significance after adjustment. Notably, patients classified as having high frailty had a 57% greater risk of in-hospital death compared with those at low frailty risk.

In our cohort, in-hospital mortality reached 19.3%. Although similar values have been described internationally, direct comparisons must be interpreted with caution due to important methodological differences across studies. For example, cohorts from Singapore (16.1%) [30] and China (17.3%) [31] were conducted strictly in the pre-pandemic period, while others applied universal COVID-19 testing, which may influence case definitions and severity profiles. Likewise, European estimates ranging from 13% to 19.3% reflect heterogeneous timeframes and population characteristics [7]. Given that our study spans pre-pandemic, pandemic, and early post-pandemic years without the ability to differentiate COVID-related from non–COVID-related pneumonias, variations in healthcare access, hospital strain, diagnostic overlap, and clinical practice during the pandemic may partially explain the higher mortality observed. Therefore, while our mortality estimate falls within the upper range of international reports, these comparisons should be interpreted with caution.

The temporal variation in in-hospital mortality observed in this study—particularly the peak recorded in 2020 and 2021—likely reflects the indirect impact of the COVID-19 pandemic on patients hospitalized with community-acquired pneumonia. During this period, diagnostic overlap between viral and bacterial pneumonias, delays in healthcare access, and the overall strain on hospital capacity may have contributed to higher mortality rates.

In this study 50.45% of patients presented an intermediate frailty risk, whereas 24.92% belonged to the high-risk group. These proportions are, to some extent, consistent with those reported by Rosario et al. (2024), who identified 47.6% of patients at high risk and 35.9% at intermediate risk of frailty in a cohort of older adults hospitalized with pneumonia [32]. Similarly, frailty assessed using the HFRS was independently associated with mortality, with patients classified as high risk showing a 57% higher risk of death compared with those at low risk (HR: 1.57; 95% CI: 1.47–1.68). These findings are consistent with the meta-analysis conducted by Yang et al. (2024), who analyzed 16 studies and reported that mortality was 2.5 times higher in the intermediate frailty group and 3.5 times higher in the high frailty group compared with non-frail patients [19]. In addition, individuals with higher frailty levels showed an increased risk of hospital readmissions and longer lengths of stay. Consistently, Zhao et al. (2023) observed a fivefold higher risk of in-hospital mortality among frail patients compared with robust ones (HR = 5.01; 95% CI: 1.51–16.57; *p* = 0.008) [16].

From a pathophysiological perspective, frailty increases the risk of mortality in patients with pneumonia due to a combination of immunological, muscular, and metabolic alterations that impair the body’s ability to respond effectively to acute infectious stress. Firstly, immunosenescence characterized by T- and B-cell dysfunction, reduced phagocytic capacity, and the expansion of senescent cells that secrete pro-inflammatory cytokines (IL-6, TNF-α, IL-1β) leads to a chronic low-grade inflammatory state which impairs the immune system’s ability to control infection and amplifies pulmonary tissue damage [5,33,34,35]. In addition, sarcopenia a central component of frailty reduces respiratory muscle strength and the cough reflex, thereby promoting secretion retention, hypoventilation, and the development of infectious complications [36,37,38]. This muscle deterioration, together with systemic inflammation, accelerates protein catabolism and limits tissue repair, creating a vicious cycle of functional decline and exaggerated inflammatory response [39]. We hypothesize that muscle deterioration, associated with alterations in mitochondrial bioenergetics, reduced ATP production capacity, and oxidative stress, plays a key role in the relationship between the risk of frailty and poor outcomes of CAP in older adults.

Moreover, frailty entails a reduction in multiorgan physiological reserve, impairing the ability of body systems to compensate for the hypoxia and oxidative stress induced by pneumonia, thereby more frequently precipitating multiorgan failure and death [14]. Finally, frail older adults more frequently present with swallowing impairments and a diminished cough reflex [37,40,41], predisposing them to aspiration pneumonia and infections caused by pathogens of greater virulence or antimicrobial resistance, as recently described in geriatric populations [42].

On the other hand, in our analysis, the Charlson Comorbidity Index lost statistical significance after adjustment for age, sex, frailty level, severity level (DRG severity), and diagnosis-related relative weight, suggesting that comorbidity alone does not independently explain the risk of mortality. Rather, its effect appears to be attenuated by the influence of frailty and other clinical determinants. Szakmany et al. (2021) demonstrated that both frailty and comorbidity are associated with mortality in patients with pneumonia, although each shows only moderate individual predictive capacity [43]. Complementarily, Gilbert et al. (2024) argue that risk stratification models for hospitalized older adults should simultaneously integrate the assessment of both frailty and comorbidities, as these dimensions reflect distinct yet complementary aspects of overall health status [44]. However, the authors also caution that the predictive capacity of these indices may be limited if other functional, clinical, and socioeconomic factors are not simultaneously considered in the prognosis of older patients [44]. In this context, our findings reinforce the need to systematically incorporate frailty assessment, but without replacing comorbidity indices, to improve risk stratification and clinical decision-making in older adults hospitalized with CAP.

In our analysis, the DRG severity level also emerged as a strong and independent predictor of in-hospital mortality, with each incremental level associated with a 66% higher hazard of death. This variable reflects the clinical and resource-based classification of hospitalization complexity used in the Chilean DRG system, integrating both diagnostic and procedural information. Its strong association with mortality highlights the relevance of administrative severity indices as complementary tools to capture acute physiological stress and treatment intensity during hospitalization. However, while DRG severity reflects the immediate clinical burden of disease, frailty captures the underlying biological vulnerability that predisposes older adults to poor outcomes. The concurrent significance of both variables in our model reinforces the notion that mortality in pneumonia results from the interaction between acute illness severity and pre-existing physiological vulnerability.

Finally, in our national cohort, advanced age and male sex emerged as independent determinants of in-hospital mortality due to pneumonia. The risk increased progressively across age groups, doubling among individuals aged 90 years or older (HR: 2.41; 95% CI: 2.27–2.56), while men showed a 10% higher risk compared with women (HR: 1.10; 95% CI: 1.06–1.14). This pattern has been previously described in the international literature. Pessoa et al. (2020) reported a 22% higher risk of death among men hospitalized with pneumonia in Portugal [9] and Ramírez et al. (2017) found higher mortality rates among men in United State cohort [6]. More recently, Gonçalves-Pereira et al. (2025) confirmed in a trend analysis covering 2009–2019 that mortality remained consistently higher in men [45]. These differences are explained, at least in part, by immunological and hormonal mechanisms, whereby men exhibit a less efficient and more pro-inflammatory immune response, predisposing them to greater severity and higher mortality in respiratory infections such as pneumonia [46]. In addition, social and cultural factors, as well as differences in habits and lifestyles, should also be considered. Likewise, advanced age emerges as an independent predictor of mortality, in line with recent evidence documenting an exponential increase in risk beyond the age of 85 years [5,7]. This increased risk is explained by biological processes such as immunosenescence and inflammation, which reduce the effectiveness of immune responses and promote more extensive tissue damage [5]. With aging, thymic involution limits the production of T lymphocytes and the ability to generate immunological memory, while dendritic cell dysfunction and a reduced antibody response impair the clearance of Streptococcus pneumoniae [5,47]. This is further compounded by a state of chronic low-grade inflammation mediated by cytokines such as IL-6, TNF-α, and NF-κB, which impairs pulmonary function and amplifies the local inflammatory response [5].

### Strengths and Limitations

Our study presents several strengths. First, we analyzed a large national cohort of 58,040 older adults hospitalized with CAP, providing robust statistical power and external validity. A substantial proportion of this population (75.4%) exhibited an intermediate or high risk of frailty according to the HFRS, allowing a detailed analysis across distinct frailty levels. Another strength lies in the use of nationwide hospital administrative data, which ensured comprehensive coverage of all public hospitals in Chile. The use of standardized diagnostic codes and structured discharge records minimizes information bias and enables reproducibility. Moreover, the application of the HFRS within administrative datasets represents a growing area of research due to its potential for automation and for informing population health management at both hospital and territorial levels. This approach is not only useful for resource allocation and planning but can also contribute to individualized, evidence-based medical decision-making. In addition, combining frailty and comorbidity indices such as the HFRS and the Charlson Comorbidity Index proved valuable for refining risk stratification in older inpatients. It could be further explored to enhance predictive accuracy in future studies.

However, some limitations should be acknowledged. First, the use of administrative data precludes access to detailed clinical variables such as functional status, nutritional condition, laboratory parameters, or radiological findings, which may influence prognosis. Second, clinical pneumonia severity scores such as the PSI and CURB-65 are not recorded in administrative datasets and were therefore unavailable in this study. Although the DRG severity level reflects hospitalization complexity and resource utilization, it does not incorporate physiological or laboratory parameters included in PSI or CURB-65, which limits direct comparability with international CAP cohorts. Third, mortality was restricted to in-hospital events, as post-discharge outcomes were not available in the database; therefore, the true long-term mortality burden may be underestimated. Fourth, although the HFRS has been validated internationally, its performance might vary across healthcare systems, and our results are based solely on the public hospital network, potentially limiting generalizability to private settings. Fifth, this analysis was restricted to hospitalizations recorded in the public healthcare system (FONASA), which covers approximately 85% of Chile’s population aged ≥60 years. Patients treated in private hospitals (ISAPRE system) were omitted, as private healthcare providers do not routinely share clinical or administrative data with the national health information system, which may limit the representativeness of the findings for the entire national hospital network. A further limitation is our inability to differentiate between pre-pandemic, pandemic, and post-pandemic hospitalizations or to distinguish COVID-19–related pneumonias from non-COVID CAP. This case-mix heterogeneity may partially influence in-hospital mortality patterns and complicates direct comparisons with studies that exclusively analyzed pre-pandemic CAP or applied systematic COVID testing. Finally, despite the large sample size and robust adjustment for confounders, residual confounding due to unmeasured factors such as socioeconomic status or system- and hospital-level care factors cannot be completely excluded.

## 5. Conclusions

In a national cohort of 58,040 older adults hospitalized with CAP, frailty emerged as a strong and independent determinant of in-hospital mortality, even after adjustment for age, sex, comorbidity, and clinical severity. Patients classified as having high frailty had a 57% higher risk of death compared with those with low frailty, confirming the prognostic value of this condition in acute hospitalization settings. These findings underscore the importance of systematically incorporating frailty assessment into risk stratification models and routine clinical practice to better identify vulnerable patients, optimize resource allocation, and support more individualized preventive and therapeutic strategies.

## Figures and Tables

**Table 1:** Baseline characteristics of the study population.

Variables	n = 58,040
Age (years) [mean (DS)]	77.82 (9.67)
Age Group	
60–69 [n (%)]	13,479 (23.22%)
70–79 [n (%)]	18,429 (31.75%)
80–89 [n (%)]	18,770 (32.34%)
≥90 [n (%)]	7362 (12,69%)
Sex	
Female [n (%)]	29,700 (51.17%)
Male [n (%)]	28,340 (48.82%)
Hospital outcome	
Survived [n (%)]	46,841 (80.70%)
Deceased [n (%)]	11,199 (19.30%)
Frailty Risk	
Low [n (%)]	14,297 (24.63%)
Intermediate [n (%)]	29,276 (50.45%)
High [n (%)]	14,467 (24.92%)
Severity level-DRG	
Mild [n (%)]	1300 (2.24%)
Moderate [n (%)]	11,233 (19.36%)
Severe [n (%)]	45,507 (78.40%)
Charlson comorbidity weight [mean (SD)]	2.11 (1.79)
Length of hospital stay (days) [mean (DS)]	8.76 (12.45)
Relative weight (Unitless) [mean (DS)]	1.22 (1.38)

**Table 2:** Unadjusted and adjusted hazard ratios for in-hospital mortality from Cox proportional hazards models.

Variable	HR (Unadjusted)	*p*	HR (Adjusted)	*p*
Age group: 70–79 (ref. 60–69)	1.33 (1.26–1.40)	<0.001	1.28 (1.22–1.35)	<0.001
Age group: 80–89 (ref. 60–69)	1.86 (1.77–1.97)	<0.001	1.74 (1.65–1.83)	<0.001
Age group: ≥90 (ref. 60–69)	2.59 (2.43–2.76)	<0.001	2.41 (2.27–2.56)	<0.001
Sex: male (ref. female)	1.04 (1.00–1.08)	0.054	1.10 (1.06–1.14)	<0.001
Frailty risk: Intermediate (ref low)	1.42 (1.37–1.48)	<0.001	1.34 (1.29–1.39)	<0.001
Frailty risk: High (ref. low)	1.77 (1.66–1.90)	<0.001	1.57 (1.47–1.68)	<0.001
Charlson comorbidity weight (per unit)	1.01 (1.00–1.02)	0.046	0.99 (0.98–1.00)	0.198
Severity level-DRG (per level)	1.63 (1.56–1.70)	<0.001	1.66 (1.58–1.73)	<0.001
Relative weight (per unit)	0.94 (0.93–0.95)	0.101	0.94 (0.93–0.95)	0.091

## Data Availability

The data that support the findings of this study are available from the corresponding author upon reasonable request.

## Abbreviations

CAP	Community-Acquired Pneumonia
DRG	Diagnosis-Related Group
FONASA	National Health Fund (Fondo Nacional de Salud, Chile)
ICD-10	International Classification of Diseases
HFRS	Hospital Frailty Risk Score
PSI	Pneumonia Severity Index

## References

[1] World Health Organization (2020). The Top 10 Causes of Death. https://www.who.int/news-room/fact-sheets/detail/the-top-10-causes-of-death.

[2] Callahan C.M., Wolinsky F.D. (1996). Hospitalization for pneumonia among older adults. J. Gerontol. Ser. A Biol. Sci. Med. Sci..

[3] Vos T., Lim S.S., Abbafati C., Abbas K.M., Abbasi M., Abbasifard M., Abbasi-Kangevari M., Abbastabar H., Abd-Allah F., Abdelalim A. (2020). Global burden of 369 diseases and injuries in 204 countries and territories, 1990–2019: A systematic analysis for the Global Burden of Disease Study 2019. Lancet.

[4] Troeger C., Blacker B., Khalil I.A., Rao P.C., Cao J., Zimsen S.R., Albertson S.B., Deshpande A., Farag T., Abebe Z. (2018). Estimates of the global, regional, and national morbidity, mortality, and aetiologies of lower respiratory infections in 195 countries, 1990–2016: A systematic analysis for the Global Burden of Disease Study 2016. Lancet Infect. Dis..

[5] Kang N., Subramanian V.S., Agrawal A. (2025). Influence of Aging and Immune Alterations on Susceptibility to Pneumococcal Pneumonia in the Elderly. Pathogens.

[6] Averin A., Shaff M., Weycker D., Lonshetyn A., Sato R., Pelton S.I. (2021). Mortality and readmission in the year following hospitalization for pneumonia among US adults. Respir. Med..

[7] Cocchio S., Cozzolino C., Furlan P., Cozza A., Tonon M., Russo F., Saia M., Baldo V. (2024). Pneumonia-related hospitalizations among the elderly: A retrospective study in Northeast Italy. Diseases.

[8] Carlos P., Gomes R., Coelho J., Chaves C., Tuna C., Louro M. (2023). CURB-65 and long-term mortality of community-acquired pneumonia: A retrospective study on hospitalized patients. Cureus.

[9] Pessoa E., Bárbara C., Viegas L., Costa A., Rosa M., Nogueira P. (2020). Factors associated with in-hospital mortality from community-acquired pneumonia in Portugal: 2000–2014. BMC Pulm. Med..

[10] Buzzo A.R., Roberts C., Mollinedo L.G., Quevedo J.M., Casas G.L., Soldevilla J.M.S. (2013). Morbidity and mortality of pneumonia in adults in six Latin American countries. Int. J. Infect. Dis..

[11] Chongthanadon B., Thirawattanasoot N., Ruangsomboon O. (2023). Clinical factors associated with in-hospital mortality in elderly versus non-elderly pneumonia patients in the emergency department. BMC Pulm. Med..

[12] Arancibia F., Andino P., Gutierrez-Arias R., Parraguez C., Astorga F. (2022). Tendencias en la mortalidad por neumonía en adultos en Chile, 2000–2016. Rev. Chil. De Enfermedades Respir..

[13] Fried L.P., Tangen C.M., Walston J., Newman A.B., Hirsch C., Gottdiener J., Seeman T., Tracy R., Kop W.J., Burke G. (2001). Frailty in older adults: Evidence for a phenotype. J. Gerontol. Ser. A Biol. Sci. Med. Sci..

[14] Clegg A., Young J., Iliffe S., Rikkert M.O., Rockwood K. (2013). Frailty in elderly people. Lancet.

[15] Huang S., Wang Y., Chen L., Chen X. (2022). Use of a frailty index based upon routine laboratory data to predict complication and mortality in older community-acquired pneumonia patients. Arch. Gerontol. Geriatr..

[16] Zhao H., Tu J., She Q., Li M., Wang K., Zhao W., Huang P., Chen B., Wu J. (2023). Prognostic significance of frailty in hospitalized elderly patients with community-acquired pneumonia: A retrospective cohort study. BMC Geriatr..

[17] Richards S.J., D’Souza J., Pascoe R., Falloon M., Frizelle F.A. (2019). Prevalence of frailty in a tertiary hospital: A point prevalence observational study. PLoS ONE.

[18] Luo J., Tang W., Sun Y., Jiang C. (2020). Impact of frailty on 30-day and 1-year mortality in hospitalised elderly patients with community-acquired pneumonia: A prospective observational study. BMJ Open.

[19] Yang Y., Zhong Y. (2024). Impact of frailty on pneumonia outcomes in older patients: A systematic review and meta-analysis. Eur. Geriatr. Med..

[20] Park C.M., Kim W., Rhim H.C., Lee E.S., Kim J.H., Cho K.H., Kim D.H. (2021). Frailty and hospitalization-associated disability after pneumonia: A prospective cohort study. BMC Geriatr..

[21] Drosdowsky A., Gough K. (2022). The Charlson Comorbidity Index: Problems with use in epidemiological research. J. Clin. Epidemiol..

[22] Canaslan K., Ates Bulut E., Kocyigit S.E., Aydin A.E., Isik A.T. (2022). Predictivity of the comorbidity indices for geriatric syndromes. BMC Geriatr..

[23] Gilbert T., Neuburger J., Kraindler J., Keeble E., Smith P., Ariti C., Arora S., Street A., Parker S., Roberts H.C. (2018). Development and validation of a Hospital Frailty Risk Score focusing on older people in acute care settings using electronic hospital records: An observational study. Lancet.

[24] Eckart A., Hauser S.I., Haubitz S., Struja T., Kutz A., Koch D., Neeser O., Meier M.A., Mueller B., Schuetz P. (2019). Validation of the hospital frailty risk score in a tertiary care hospital in Switzerland: Results of a prospective, observational study. BMJ Open.

[25] Hoogendijk E.O., Afilalo J., Ensrud K.E., Kowal P., Onder G., Fried L.P. (2019). Frailty: Implications for clinical practice and public health. Lancet.

[26] Roe L., Normand C., Wren M.-A., Browne J., O’Halloran A.M. (2017). The impact of frailty on healthcare utilisation in Ireland: Evidence from the Irish longitudinal study on ageing. BMC Geriatr..

[27] Imam T., Konstant-Hambling R., Fluck R., Hall N., Palmer J., Conroy S. (2021). The hospital frailty risk score—Outcomes in specialised services. Age Ageing.

[28] Charlson M.E., Pompei P., Ales K.L., MacKenzie C.R. (1987). A new method of classifying prognostic comorbidity in longitudinal studies: Development and validation. J. Chronic Dis..

[29] Quan H., Sundararajan V., Halfon P., Fong A., Burnand B., Luthi J.-C., Saunders L.D., Beck C.A., Feasby T.E., Ghali W.A. (2005). Coding algorithms for defining comorbidities in ICD-9-CM and ICD-10 administrative data. Med. Care.

[30] Zhang Z.X., Yong Y., Tan W.C., Shen L., Ng H.S., Fong K.Y. (2018). Prognostic factors for mortality due to pneumonia among adults from different age groups in Singapore and mortality predictions based on PSI and CURB-65. Singap. Med. J..

[31] Hao Y., Zhang H., Yan Y., Zhu Y., Zhang F. (2022). A Model to Predict In-hospital Mortality in Elderly Patients with Community-acquired Pneumonia: A Retrospective Study. medRxiv.

[32] Rosario B.H., Quah J.L., Chang T.Y., Cantiller Barrera V., Lim A., Sim L.E., Conroy S., Dhaliwal T.K. (2024). Validation of the Hospital Frailty Risk Score in older adults hospitalized with community-acquired pneumonia. Geriatr. Gerontol. Int..

[33] Yanagi S., Tsubouchi H., Miura A., Matsuo A., Matsumoto N., Nakazato M. (2017). The impacts of cellular senescence in elderly pneumonia and in age-related lung diseases that increase the risk of respiratory infections. Int. J. Mol. Sci..

[34] Liu Z., Liang Q., Ren Y., Guo C., Ge X., Wang L., Cheng Q., Luo P., Zhang Y., Han X. (2023). Immunosenescence: Molecular mechanisms and diseases. Signal Transduct. Target. Ther..

[35] Theodorakis N., Feretzakis G., Hitas C., Kreouzi M., Kalantzi S., Spyridaki A., Kollia Z., Verykios V.S., Nikolaou M. (2024). Immunosenescence: How aging increases susceptibility to bacterial infections and virulence factors. Microorganisms.

[36] Morisawa T., Kunieda Y., Koyama S., Suzuki M., Takahashi Y., Takakura T., Kikuchi Y., Matsuda T., Fujino Y., Sawa R. (2021). The relationship between sarcopenia and respiratory muscle weakness in community-dwelling older adults. Int. J. Environ. Res. Public Health.

[37] Okazaki T., Suzukamo Y., Miyatake M., Komatsu R., Yaekashiwa M., Nihei M., Izumi S., Ebihara T. (2021). Respiratory muscle weakness as a risk factor for pneumonia in older people. Gerontology.

[38] Kera T., Kawai H., Obuchi S. (2025). Respiratory Sarcopenia: Current Understanding of Concepts and Future Issues. J. Am. Med. Dir. Assoc..

[39] Nagano A., Wakabayashi H., Maeda K., Kokura Y., Miyazaki S., Mori T., Fujiwara D. (2021). Respiratory sarcopenia and sarcopenic respiratory disability: Concepts, diagnosis, and treatment. J. Nutr. Health Aging.

[40] Chen K.-C., Lee T.-M., Wu W.-T., Wang T.-G., Han D.-S., Chang K.-V. (2021). Assessment of tongue strength in sarcopenia and sarcopenic dysphagia: A systematic review and meta-analysis. Front. Nutr..

[41] Fujishima I., Fujiu-Kurachi M., Arai H., Hyodo M., Kagaya H., Maeda K., Mori T., Nishioka S., Oshima F., Ogawa S. (2019). Sarcopenia and dysphagia: Position paper by four professional organizations. Geriatr. Gerontol. Int..

[42] Putot A., Garin N., Rello J., Prendki V. (2025). Comprehensive management of pneumonia in older patients. Eur. J. Intern. Med..

[43] Szakmany T., Hollinghurst J., Pugh R., Akbari A., Griffiths R., Bailey R., Lyons R.A. (2021). Frailty assessed by administrative tools and mortality in patients with pneumonia admitted to the hospital and ICU in Wales. Sci. Rep..

[44] Gilbert T., Cordier Q., Polazzi S., Street A., Conroy S., Duclos A. (2024). Combining the hospital frailty risk score with the charlson and elixhauser multimorbidity indices to identify older patients at risk of poor outcomes in acute care. Med. Care.

[45] Gonçalves-Pereira J., Froes F., Pereira F.G., Diniz A., Oliveira H., Mergulhão P. (2025). Community-acquired pneumonia mortality trends according to age and gender: 2009 to 2019. BMC Pulm. Med..

[46] Takahashi T., Iwasaki A. (2021). Sex differences in immune responses. Science.

[47] Häder A., Köse-Vogel N., Schulz L., Mlynska L., Hornung F., Hagel S., Teichgräber U., Lang S.M., Pletz M.W., Le Saux C.J. (2023). Respiratory infections in the aging lung: Implications for diagnosis, therapy, and prevention. Aging Dis..

